# A tug-of-war over the mid-latitudes

**DOI:** 10.1038/s41467-019-13714-0

**Published:** 2019-12-09

## Abstract

The amplified warming of the Arctic in recent decades has been related to extreme weather events over the mid-latitudes, but its relative importance compared to other influences is not yet well understood. A Nature Research collection highlights evidence from theoretical and observational studies, as well as implications for future extreme events.

The Arctic is currently warming faster than most parts of the world as a consequence of human greenhouse gas emissions, resulting in extensive sea-ice and snow cover decline. Although these changes play a prominent role in the public debate on climate change, they are often perceived as remote, with little direct impact on humans. On the contrary, research has suggested that amplified Arctic warming can cause changes in the atmospheric circulation associated with an increasing frequency and intensity of extreme weather events over the mid-latitudes^1^. As a consequence, amplified Arctic warming has direct economic and societal impacts for the societies in Europe, Asia and North America. However, although the existence of a link between the Arctic and mid-latitudes is now well established, there is a growing debate between scientists about the strength of the influence of Arctic warming versus that of other regions, like the Tropics, on the mid-latitudes. A new Nature Research collection from Nature Communications, Nature, Nature Geoscience and Nature Climate Change brings together Review articles and recent research on the relevance of Arctic warming for mid-latitude weather extremes.

When observational data showed an acceleration of Arctic warming and sea-ice loss from the 1990s onward, a correlation with increasing winter and summer extreme events in the mid-latitudes was noted^1^. At the time, a possible Arctic influence on the mid-latitudes was not only argued for by concurrence of trends, but also by a theoretical understanding of how polar conditions can influence lower latitudes. A decline in sea-ice and snow cover is expected to change large scale patterns of sea level pressure that influence weather conditions over the Northern hemisphere, along with changes in the latitude and strength of the fast flowing air masses of the jet stream while promoting increasingly persistent weather patterns (Fig. 1).

**Fig. 1:**
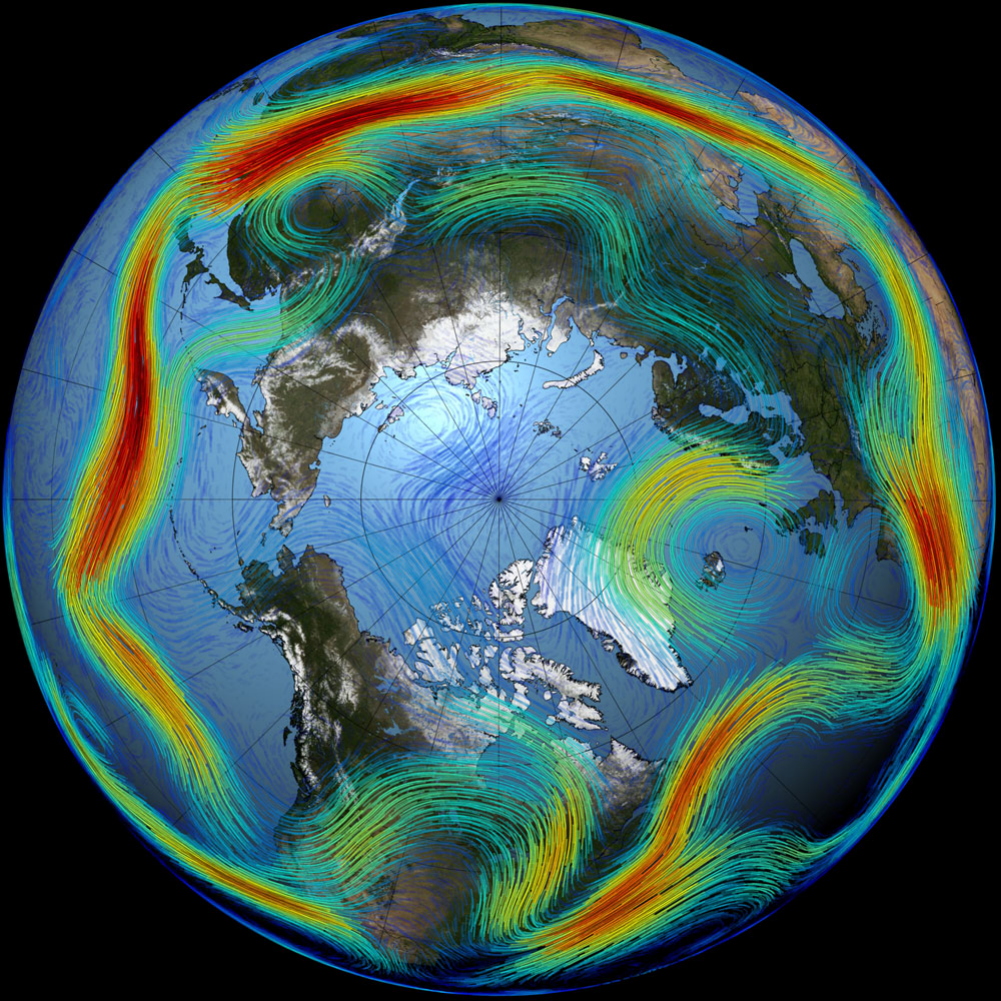
Arctic sea ice and the mid-latitude jet stream. The mid-latitude jet stream, shown here in red, is one of the components of the atmospheric circulation that is expected to change with Arctic amplification and decreasing sea ice. For example, the reduced temperature gradient between the two latitudinal bands can cause a weaker and more meandering jet stream. This causes increasingly persistent weather patterns, which in turn can result in extreme events. [Image from NASA/Goddard Space Flight Center Scientific Visualization Studio].

Possible consequences from such changes include more frequent and severe cold spells, heavy rainfalls and heat waves. Events such as the Eurasian heat wave of 2010 and the increasing frequency of winter cold extremes over East Asia and North America have been attributed to the effects of Arctic warming. The heat wave of 2010 is estimated to have caused more than 50,000 deaths in Russia alone^2^ and the economic cost of a single cold event in the US can be up to 3 billion Dollars^3^. Due to these vast societal impacts, scientific research on possible causes of single extreme weather events and their relationship to anthropogenic climate change has received significant media and public attention.

While a potential linkage between Arctic warming and mid-latitude extreme events is now generally recognized, more research is still required to evaluate the strength of the Arctic influence. Some studies have reported effects which were predicted by Artic warming, for example a weakening of the jet stream, but some scientists debated the statistical significance of these changes^4^. Furthermore, climate models, the main instruments to study causal relationships in the climate system, have shown a differing response of the atmospheric circulation to Arctic sea ice loss^5^. Such differing results could be explained by the short timeframe of observational data, with satellite measurements starting only in 1979, which is too short to detect significant trends. It is also possible that climate models may differ in representations of key processes that influence their response to sea ice variations^5^. These factors might play a role, but the conflicting evidence more likely reflects the multitude and complexity of influences on mid-latitude conditions from more than just the Arctic.

This complexity has given rise to a tug-of-war between contrasting effects of the Arctic and Tropics on the mid-latitudes. It is well established that the Arctic is not the only region that changes atmospheric circulation, and that other regions, in particular the Tropics, can exert an influence over the mid-latitudes as well. Like the Arctic, the Tropics are also seeing dramatic changes due to anthropogenic greenhouse gas emissions. There is growing evidence to suggest that the Tropics could counter some of the effects a warming Arctic has on atmospheric circulation^4^. For example, the atmosphere exhibits its strongest warming at different heights in the Arctic and the Tropics and as a consequence of this asymmetry, the jet stream is expected to shift southward in response to Arctic and northward in response to Tropical warming^4^. However, the competing effects of the regions do not necessarily cancel each other out, and therefore identifying which region is the strongest influence is crucial for unlocking how the mid-latitudinal weather changes due to global warming. Due to the complexity in reaching a consensus on how the mid-latitudes climate will change in the future, this demands collaborative research efforts, for example with systematic model comparison projects.

The degree to which the consequences of anthropogenic warming in the Arctic and Tropics controls extreme weather events at the mid-latitudes will have significant implications for future climate risk assessment in these regions. Of course, the most efficient way to prevent catastrophic changes anywhere would be to reduce emissions that are driving the changes in the Arctic and the Tropics. If these efforts are not successful, however, a consistent and reliable estimation of expected extreme weather events is vital for governments to allow an effective adaptation to climate risks, as well as for stakeholders, such as the energy and insurance sectors.

In addition to the direct political and economic interest, there are also societal implications of this research, as it has the potential to increase awareness of global climate change in those societies living in the high-population and high-emission regions of the mid-latitudes. Currently, some of the strongest effects of climate change are observed in the high- and low-latitudes and hence primarily affect societies around the Arctic Ocean or on Tropical Island states, which are often marginalized in the debates on climate change. The mechanisms discussed in the Nature Research Collection demonstrate once again that changes happening at distant locations cannot be viewed in isolation, but that the consequences of human greenhouse gas emissions affect all societies.
